# Organization of the Human Frontal Pole Revealed by Large-Scale DTI-Based Connectivity: Implications for Control of Behavior

**DOI:** 10.1371/journal.pone.0124797

**Published:** 2015-05-06

**Authors:** Joseph M. Orr, Harry R. Smolker, Marie T. Banich

**Affiliations:** 1 Institute of Cognitive Science, University of Colorado Boulder, Boulder, Colorado, United States of America; 2 Department of Psychology and Neuroscience, University of Colorado Boulder, Boulder, Colorado, United States of America; University College London, UNITED KINGDOM

## Abstract

The goal of the current study was to examine the pattern of anatomical connectivity of the human frontal pole so as to inform theories of function of the frontal pole, perhaps one of the least understood region of the human brain. Rather than simply parcellating the frontal pole into subregions, we focused on examining the brain regions to which the frontal pole is anatomically and functionally connected. While the current findings provided support for previous work suggesting the frontal pole is connected to higher-order sensory association cortex, we found novel evidence suggesting that the frontal pole in humans is connected to posterior visual cortex. Furthermore, we propose a functional framework that incorporates these anatomical connections with existing cognitive theories of the functional organization of the frontal pole. In addition to a previously discussed medial-lateral distinction, we propose a dorsal-ventral gradient based on the information the frontal pole uses to guide behavior. We propose that dorsal regions are connected to other prefrontal regions that process goals and action plans, medial regions are connected to other brain regions that monitor action outcomes and motivate behaviors, and ventral regions connect to regions that process information about stimuli, values, and emotion. By incorporating information across these different levels of information, the frontal pole can effectively guide goal-directed behavior.

## Introduction

Neuroanatomy has long provided insights into the functional organization of the human brain, from the distinctions made by Brodmann over 100 years ago [1] to mapping of the visual cortex in the 70s and 80s [2–4]. Such insights have also been made for regions of the frontal lobe of the brain, such as work distinguishing between dorsal and ventral regions of mid-lateral prefrontal cortex [5,6]. In that tradition, the current report focuses on what can be surmised with regards to the functions of the frontal pole by virtue of examining its anatomical connections.

There is clear consensus that the frontal pole plays a critical role in higher-order cognition [7], yet, there is little consensus with regards to a detailed theory of its functioning. One hypothesis, the gateway hypothesis, suggests that the frontal pole (FP) acts as a supervisory attentional control system that selects between attending to internal (‘stimulus-independent’) information, or attending to external (‘stimulus-oriented’) information [8,9]. According to this hypothesis, the lateral division of the FP appears to respond to task conditions where participants must attend to or process internally maintained information, such as task goals, whereas the medial division responds when participants attend to external stimuli [10,11], which must be tracked to ensure that the current goal is consistent with external conditions.

Support for a medial-lateral distinction of the FP is further supported by examining patterns of task-related functional co-activation of the FP. The lateral FP co-activates with regions known to be involved in top-down control such as the dorsolateral prefrontal cortex (DLPFC), the anterior cingulate cortex (ACC), anterior insula and lateral parietal cortex [12]. In contrast, the medial FP is co-activated with the temporal pole, posterior cingulate, and posterior superior temporal sulcus. While these regions are typically thought of as belonging to the ‘default mode’ or ‘task negative’ network [13,14], Gilbert and colleagues showed that these regions, in conjunction with the medial FP, respond during mentalizing, or inferring the state of another agent, an arguably at least somewhat externally focused task [12]. A recent investigation into the cytoarchitecture of the human FP by Bludau and colleagues revealed evidence for distinct medial and lateral cytoarchitectonic areas, which they labeled Fp2 and Fp1, respectively [15], further supporting the medial-lateral distinction in FP organization. Bludau and colleagues also examined the patterns of functional co-activation of these regions as assessed with fMRI and found that they co-activate with different brain regions in a manner similar to that reported by Gilbert and colleagues (2010). To our knowledge, Bludau and colleagues are the first to show evidence for a medial-lateral difference in cytoarchitecture of the human FP. While Brodmann’s original demarcation of the frontal pole (i.e., area 10) did not extend to the orbital surface (which he labeled area 11 [1], both Bludau and colleagues [15] and Ongür, Ferry, & Price [16] showed that the orbital surface was more similar in cytoarchitecture to BA10 than BA11.

However, O’Reilly [17] has suggested that the prefrontal cortex (PFC) in additional to displaying a medial-lateral distinction in functional organization also exhibits a ventral-dorsal gradient as well. In this scheme, the medial PFC represents value and motivation (i.e., *Hot* cognition) whereas lateral PFC represents more cold cognitive calculations. The ventral PFC represents semantic information whereas the dorsal PFC represents action-related information (e.g., stimulus-response mappings). While there is strong evidence of the FP exhibiting such a medial-lateral gradient, it is unclear whether a ventral-dorsal gradient exists in the FP. Given the large size of the FP and its position at the intersection of the ventral and dorsal PFC streams, it seems sensible to predict that such a gradient also exists within the FP.

One way that we can gain insight in to how exactly the FP operates is to delineate the regions to which it connects. While previous studies have examined the brain regions that show functional co-activation with the FP [12,15], little is known about the anatomical connections of the FP, an issue directly addressed in the present study. Even in animals, only a small number of studies have examined the connections of this region. A tracer study with monkeys has suggested 3 subregions of the FP: a lateral part, a medial part, and an orbital part [18]; the lateral part of FP connects to the DLPFC and superior temporal regions, the medial part connects to the anterior and posterior cingulate via the cingulum bundle, and the orbital part connects to temporal pole and the amygdala via the uncinate fasciculus. These findings were largely confirmed by a recent study of human connectivity via Diffusion Tensor Imaging (DTI) [19]. Given that the human FP is the most evolutionarily advanced brain region [20], it is crucial to better understand the connectivity of the human FP.

Here we follow in using patterns of DTI connectivity to provide insights into the parcellation of other brain regions, such as has been done for the cingulate [21], the SMA and pre-SMA [22,23], insula [23], and a prior, limited study of the FP [19]. However, most of these prior studies have included low numbers of participants, typically around 10. As part of a large-scale effort to examine the psychological and neural mechanisms of executive function, we collected data on close to 100 participants. This ten-fold increase in the number of participants from what is typically recruited enabled us to more closely examine how patterns of connectivity within the FP vary across participants, including the stability of clusters and consistency of tracts. Furthermore, we took advantage of the availability of ultra-high resolution data freely provided by the Human Connectome Project (HCP; http://humanconnectome.org). A limited set of HCP data was analyzed as a supplemental analysis in order to provide confirmation of the tractography results obtained in our original sample. By examining both clustering of regions within the FP as well as connectivity, we are able to provide, in the discussion, a putative organization of this important brain region.

## Methods

### Participants

A total of 100 participants were scanned. However, due to technical issues, the first 7 participants were scanned with a different sequence (i.e., 71 directions collected in two orthogonal runs instead of one run), so these participants were excluded. Thus, data from the remaining 93 participants were analyzed (51 female, mean age 20.6 years). All participants were either undergraduate or graduate students at the University of Colorado (CU) Boulder. Participants were recruited through an online, CU-Boulder-based recruitment website, and were paid for their participation. Written informed consent was obtained prior to both experimental sessions and all experimental protocols were approved by CU-Boulder’s Institutional Review Board prior to data collection. All participants were right-handed.

In addition, data from 30 participants from the Human Connectome Project (HCP; http://humanconnectome.org) were analyzed. Participants were selected from the WU-Minn Young Adult HCP Dataset S500 release. Mean age was 28.7 years (range: 22–35 years) with 13 male participants. While some of the participants were part of a twin pair (15 twins), none of the included participants were related.

### Imaging Acquisition

Magnetic resonance imaging (MRI) of the brain was acquired on each subject using a Siemens 3-Tesla Magnetom TIM Trio MRI scanner (Siemens AG, Munich, Germany) with a 12-channel head coil. Structural images were acquired with a T1-weighted 3D magnetization prepared rapid gradient multi-echo sequence (MPRAGE; sagittal plane; repetition time [TR] = 2530 ms; echo times [TE] = 1.64 ms, 3.5 ms, 5.36 ms, 7.22 ms, 9.08 ms; GRAPPA parallel imaging factor of 2; 1 mm^3^ isomorphic voxels, 192 interleaved slices; FOV = 256mm; flip angle = 7°; time = 6:03 min). Structural connectivity was assessed with a diffusion weighted scan [71 gradient directions; TR = 9600 ms; TE = 86 mm; GRAPPA parallel imaging factor 2; β-value = 1000 s/mm^2^; FOV = 256 mm; 72 slices; 2 mm^3^ isomorphic voxels; 7 β0 images].

Data included from the HCP database consisted of high-resolution structural images (T1 and T2 weighted scans) and diffusion images, collected on a specially modified Siemens Skyra 3T scanner, over 4 total imaging sessions. The development of the sequences used in the HCP are discussed in detail elsewhere [24–26]. T1-weighted images were collected with a 3D-MPRAGE sequence (sagittal plane; TR = 2400 ms; TE = 2.14 ms; TI = 1000 ms; iPAT = 2; 0.7 mm isotropic voxels, 256 interleaved slices; FOV = 224 mm; flip angle = 8°; time = 7:40 min) and T2-weighted images were collected with a T2-SPACE sequence (Sampling Perfection with Application optimized Contrasts using different flip angle Evolutions; TR = 3200; TE = 565 ms; iPAT = 2; 0.7 mm isotropic voxels, 256 interleaved slices; FOV = 224 mm; variable flip angle; time = 8:24 min). Two sets of T1 and T2 weighted images were collected and were averaged during preprocessing, as discussed in more detail below. Diffusion images consisted of 6 separate runs representing 3 different sets of monopolar diffusion weighted directions (90 directions each, plus 6 b = 0 acquisitions), with each set acquired once with right-to-left and left-to-right phase encoding polarities. Diffusion weighting consisted of 3 shells of b = 1000, 2000, and 3000 s/mm^2^. All diffusion runs were acquired with the following parameters: TR = 5520 ms; TE = 89.5 ms; flip angle = 78°; 111 slices; 1.25 isotropic voxels; multiband factor = 3).

### Imaging Preprocessing

Diffusion weighted images collected for this study were pre-processed using FSL’s FDT toolbox. Images were corrected for motion and eddy current distortions. Data from the HCP database were downloaded in a preprocessed form. The images had undergone the minimal preprocessing pipeline (v. 3.1) as described elsewhere [27].

Briefly, the structural images first went through the *PreFreeSurfer* pipeline which performed gradient distortion correction, alignment and averaging of the two sets of T1w and T2w scans, brain extraction, readout distortion correction, bias field correction, then registration to MNI space. The *FreeSurfer* pipeline consisted of a custom version of FreeSurfer v. 5.3 designed to perform a more robust brain extraction and more accurate mapping of the white and pial surfaces. The *PostFreeSurfer* pipeline converts the FreeSurfer cortical parcellations to native-space GIFTI surface meshes, and then registers the meshes to the Conte69 population average surface. These meshes are then downsampled from the 164k_fs_LR mesh to the lower resolution 32k_fs_LR mesh. Finally, the 32k_fs_LR is transformed from MNI space back to native space.

The HCP diffusion images were processed with the *Diffusion Preprocessing* pipeline. After intensity normalization, the b = 0 images of both phase encoding directions (i.e, left-to-right and right-to-left) were used to calculate EPI susceptibility-induced field distortions, which were modeled using the *eddy* tool and then corrected. The b = 0 image is then registered to the T1w, and the corrected images were resampled to 1.25 mm native space.

### Seed Masks

Masks for the left and right FP were created from the cytoarchitectonic maps of Bludau and colleagues [15]. These maps were in 2mm space. Probabilistic maps for Fp1 and Fp2 were thresholded at 50% and combined, separately for the left and right hemisphere. This resulted in a mask containing 2728 voxels in the left hemisphere and 2440 voxels in the right hemisphere.

A separate set of masks was defined for use in the HCP data analyses. The white matter surface on the 164k_fs_LR standard space mesh was converted to a volume and was then masked with the Bludau FP masks in order to create a FP surface mask. Again, separate masks were created for the left and right hemisphere.

### Imaging Data Analysis

Probability distributions of fiber orientation were then estimated at each voxel [28]. Fiber orientations were estimated in two directions for the data collected for this study, and in three directions for the HCP data. Further, for the HCP data a multi-shell model was used [29], and Rician noise replaced the default Gaussian noise assumption. Probabilistic tractography was performed between the FP masks/ surfaces and a mask of the rest of the brain (excluding left and right FP). The tractography script was provided with non-linear transformation between standard space (i.e., the MNI template on which the FP mask was defined) and each individual’s native diffusion space. Tractography data were stored in standard space in order to compare results across participants. The FP to whole-brain tractography results were stored as a connectivity matrix between each seed and target voxel, which was downsampled to 5 mm^3^ isotropic voxels to decrease storage demands. This matrix was transformed in to a cross-correlation matrix. For the volumetric mask tractography, but not the surface-based tractography, the cross-correlation matrices were Fisher-Z transformed and averaged across the 93 participants. Although the surfaces were all masked by the Bludau FP, the surfaces were different for each participant and did not contain the same number of voxels, therefore the cross-correlation matrices were not averaged.

The cross-correlation matrices were entered in to a *k*-means clustering algorithm available for the Python programming language [30]. This algorithm minimizes the squared Euclidian distance of each point to the cluster centroid. Clustering underwent 1000 replicates, each with a different set of cluster centroid locations; the solution that minimized the sum of all within-cluster point-to-centroid distances was chosen by the algorithm. We evaluated cluster solutions with *K* = 2 … 8 clusters. Clustering was done separately for each hemisphere of the FP.

We examined several clustering metrics in order to evaluate the group-level cluster solutions. In order to identify the optimal *K* clustering solution, we used the variation of information (VI) metric [31]. This metric reflects the degree of cluster overlap between two clusterings, and has been used by previous parcellation studies to select the optimal *K* [32,33]. We used a split-half procedure, where participants were divided in to two random groups, so that the clusterings for each group could be compared. The optimal *K* was defined as the smallest *K* for which VI did not increase (i.e., decreased overlap between clusterings) relative to *K*-1. Over 100 repetitions, we randomly assigned our participants to one of two groups (*N* = 46, *N* = 47, respectively), and computed the VI metric for each *K* in order to assess the overlap in clusters between the two groups. Briefly, the VI metric contains information about the similarity of the cluster solutions between the two groups (i.e., cluster solution *C* and *C’*, respectively). VI is defined as:

$$VI{\left(C,C_{K}^{\prime }\right)}_{K}=H{\left(C\right)}_{K}+H{\left(C\prime \right)}_{K}-2I{\left(C|C^{\prime }\right)}_{K}$$ (1)

where *H(C)*_*K*_ and *H* (*C*′)_*K*_ represent the amount of information of clustering C and C′, and *I(C|C*′)_*K*_ represents how much mutual information is contained in the clusterings, defined as:

$$\;\;I{\left(C,C\prime \right)}_{K}=\overset{K}{\underset{k=1}{\sum }}\overset{K\prime }{\underset{k=1}{\sum }}P\left(k,k^{\prime }\right)\cdot \log\frac{P\left(k,k^{\prime }\right)}{P\left(k\right)P\left(k^{\prime }\right)},$$ (2)

and

$$H{\left(C\right)}_{K}=\;-\;\overset{K}{\underset{k=1}{\sum }}P\left(k\right)\;\cdot \;\log P\left(k\right).$$ (3)

*P(k)* and *P(k′)* are the probability that a voxel belongs to a cluster k or cluster *k′*, respectively; i.e., the number of voxels in *k* divided by the total number of voxels in the frontal pole seed mask. *P(k*,*k′)* is the probability that a voxel belongs to cluster *k* in *C* and cluster *k′* in *C′*, calculated as the number of voxels in common for cluster *k* and cluster *k′* divided by the total number of voxels in the frontal pole seed mask. A VI of 0 means there is complete overlap of the clusterings and high values mean there is low similarity between the clusterings (bounded by log(*n*), which, given 93 participants would be 1.96). For each *K* (*K* = 2,3 … 10), we repeated the randomization procedure 100 times in order to generate confidence intervals around the mean VI so that difference in VI across *K*-values could be evaluated using paired *t*-tests.

Next, we evaluated the hierarchical nature of the different *K* solutions with the hierarchy index [33]. This index reflects the extent to which each additional cluster (e.g., from *K* = 2 to *K* = 3) stemmed from only one parent cluster. The hierarchy index for each *K* solution was computed according to:

$$HI_{K}=\frac{1}{K}\overset{K}{\underset{i=1}{\sum }}\frac{max_{j}\left(x_{ij}\right)}{\bar{x_{i}}},$$ (4)

Where

$$\;\;{\bar{x}}_{i}=\;\overset{K-1}{\underset{j=1}{\sum }}x_{ij},$$ (5),

and for each *K*, *x*_*ij*_ reflects the voxels in each cluster stemming from a cluster in solution *K*—1.

We also examined the symmetry of the cluster solutions between the left and right hemispheres. Prior studies have demonstrated high symmetry for the FP with respect to functional co-activation patterns [12,15], connectivity clusters [19], as well as cytoarchitecture [15]. Symmetry is defined as the proportion of overlap between homologous clusters of each hemisphere. As the size of the left and right FP masks were of different sizes, the symmetry calculation only included voxels present in both hemispheres.

At present, surface-based parcellations (compared to standard-space volumetric parcellations) rely more on subjective measures in order to determine the optimal clustering solution. Because the surfaces are largely 2-D and are based on individual patterns of cortical folding, the surfaces are difficult to align to the same template and do not contain the same number of voxels. Because of these issues, surface-based parcellations do not permit more objective cluster metrics and resulting statistics. To deal with this issue, previous surface-based connectivity parcellations have examined the degree of variation across participants in the center-of-gravity of each cluster and the degree of overlap in tracts contained within a cluster across participants [34]. For display and comparison purposes, we generated cluster overlap maps by dilating the surface for each participant by a 2mm radius spherical kernel. This approach made the clusters easier to average across participants so as to enable an examination of the degree to which the clusters overlapped across participants.

In order to assess the unique connectivity of each cluster for a given solution, we used the entire cluster as a seed mask for tractography with the rest of the brain. These tracts were normalized by the number of total possible streamlines, thresholded at 1% to remove excess noise, binarized, and then averaged across subjects in order to generate maps of tract overlap. Tracts were only considered if they were present in at least 50% of participants. In order to compare the cluster tracts to each other, we examined the normalized and thresholded (but not binned) cluster tractography maps. Across all participants, we compared the tracts of a given cluster to the tracts of all other clusters. A voxelwise permutation test was performed in FSL’s *randomise* using the Threshold-Free Cluster Enhancement algorithm to provide cluster-level statistics, corrected to a Family-Wise Error rate of *p*<.01; for each contrast underwent 1000 permutations.

### Functional Co-Activation

In order to gain additional information about the functional organization of the FP, we took a different approach and examined functional co-activation of the different FP clusters using the *Neurosynth* database (http://neurosynth.org) [33]. At the time of the analysis (December 2014), Neurosynth contained 347911 activations across 9721 fMRI studies that were selected without regard for the psychological process under investigation. Recent papers have used resting-state connectivity to perform functional parcellations of the FP as a complement to DTI-based structural parcellations [19,34]. In contrast, Neurosynth performs large-scale meta-analytic co-activation with a seed coordinate across all of the studies in the database. Neurosynth builds the co-activation maps by extracting activation data from any study showing activation within a 10 mm radius sphere. This approach is similar to that of Gilbert and colleagues [12], who examined patterns of functional co-activation between medial vs. lateral FP and the rest of the brain. Gilbert and colleagues identified 164 studies from a PubMed search for fMRI or PET studies mentioning a term related to the frontal pole. On the contrary, Neurosynth takes in to account activations in all 9721 studies in the database in generating co-activation maps, rather than just selecting the studies that activated the region of interest. A two-way chi-square tests for the specificity of an activation pattern by contrasting the studies that did not activate an area and the studies that did activate an area. This approach allow for stronger inferences than typical meta-analysis studies where studies are selected for activation of a specific region, as the statistics control for the prior probability of activation of a given voxel.

We created co-activation maps for each cluster in the optimal clustering solutions (i.e., K = 6 in the left hemisphere and K = 4 in the right hemisphere). We entered the center-of-gravity coordinates for each cluster (coordinates reported in Table 1) in to Neurosynth, and studies were included if they activated at least 10% of voxels in a 10 mm radius sphere around the coordinates [35]. Co-activation maps were corrected to a False-Discovery Rate of *p*<.00005, in line with previous studies publishing Neurosynth co-activation results [36].

**Table 1:** Coordinates for center-of-mass of clusters from each of the k-means parcellations.

Left Hemisphere						Right Hemisphere					
K	cluster	color	x	y	z	K	cluster	color	x	y	z
2	1	green	-15	64	14	2	1	green	15	65	13
	2	red	-11	62	-7		2	red	11	64	-8
3	1	green	-14	63	18	3	1	green	12	64	18
	2	blue	-15	65	4		2	blue	18	65	4
	3	red	-9	62	-10		3	red	9	64	-10
4	1	green	-16	62	19	4	1	green	13	64	19
	2	blue	-11	66	11		2	blue	10	66	8
	3	yellow	-16	64	1		3	yellow	25	64	2
	4	red	-9	62	-11		4	red	9	64	-9
5	1	green	-15	62	20	5	1	green	12	64	20
	2	blue	-11	66	12		2	blue	12	67	10
	3	yellow	-6	62	1		3	yellow	25	64	2
	4	red	-9	62	-12		4	red	9	64	-12
	5	orange	-22	64	1		5	orange	7	64	0
6	1	green	-15	62	20	6	1	green	12	64	20
	2	blue	-11	66	12		2	blue	12	67	10
	3	purple	-7	63	2		3	purple	8	64	1
	4	red	-4	57	-10		4	red	4	59	-11
	5	orange	-13	65	-10		5	orange	13	67	-10
	6	yellow	-24	63	2		6	yellow	26	63	2
7	1	green	-14	62	21	7	1	green	11	64	21
	2	blue	-12	66	14		2	blue	11	67	10
	3	purple	-7	64	4		3	purple	7	64	1
	4	violet	-4	57	-9		4	violet	4	60	-11
	5	red	-11	65	-10		5	red	11	67	-10
	6	orange	-18	63	-6		6	orange	22	65	-3
	7	yellow	-24	62	4		7	yellow	26	62	6
8	1	green	-12	62	22	8	1	green	12	64	21
	2	blue	-9	66	11		2	blue	5	64	13
	3	purple	-7	63	2		3	purple	7	64	1
	4	violet	-4	57	-10		4	violet	4	59	-11
	5	red	-11	65	-11		5	red	12	67	-11
	6	orange	-19	63	-6		6	orange	22	65	-3
	7	orange-yellow	-24	62	3		7	orange-yellow	17	68	8
	8	yellow	-18	65	15		8	yellow	27	61	6

Coordinates are in MNI space. Color refers to cluster labels in Fig 2.

## Results

### FP to Whole Brain Connectivity Parcellation

#### Cluster Metrics

**Fig 1:**
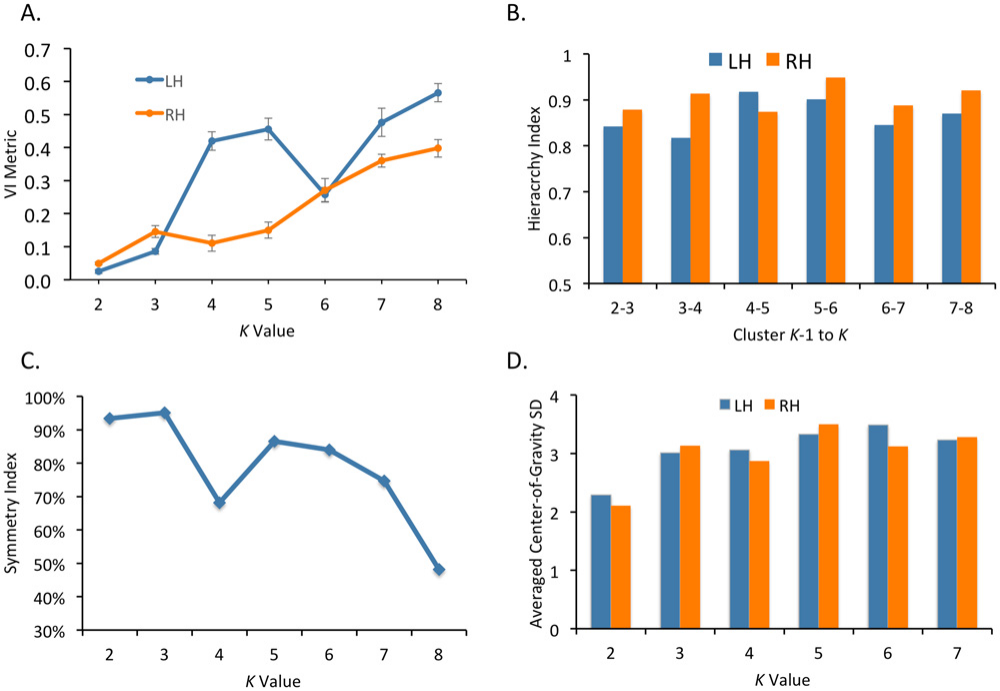
Cluster metrics. A) Variation of information metric for each *K* value and each hemisphere. Error bars represent 95% confidence intervals. B) Hierarchy index for each *K* value and each hemisphere. C) Symmetry measurement between the left and right hemispheres for each *K* value. D) Average of the standard deviation in the center-of-gravity across clusters for each *K* value.

Cluster metrics are shown in Fig 1. Following Kahnt and colleagues [33], we defined the optimal *K* as the smallest *K* greater than 2 for which VI did not significantly increase relative to *K*—1. In the left hemisphere this point was *K* = 6 (VI: 0.26), as VI significantly decreased from *K* = 5 (VI: 0.46; *t*(99) = -11.6, *p*<.001). In the right hemisphere this point was *K* = 4 (VI: 0.11), as VI significantly decreased from *K* = 3 (VI: 0.15; *t*(99) = -2.8, *p*<.005). In the left hemisphere, the hierarchy index peaked at the transition from K = 4 to K = 5, and in the right hemisphere, the hierarchy index peaked at the transition from K = 5 to K = 6. This means that at these values of *K*, additional clusters stemmed from fewer parent clusters than at low and high values of *K*. The symmetry index tended to decrease from low to high values of *K* (with the exception of a large dip at *K* = 4), meaning that as *K* increased, the symmetry of the clustering solutions between the hemispheres decreased. In order to evaluate the clusters from the HCP data, we examined the standard deviation of the cluster center-of-gravity, following [34].

#### Cluster Tracts

**Fig 2:**
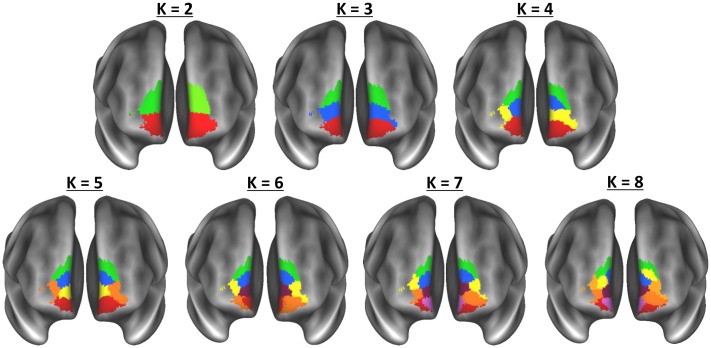
Cluster maps for all clustering solutions for the regions of cytoarchitectonic regions identified by Bludau et al. (2013) as frontal pole. Colors used here correspond to the colors of the labels and/or tracts in the following figures.

**Fig 3:**
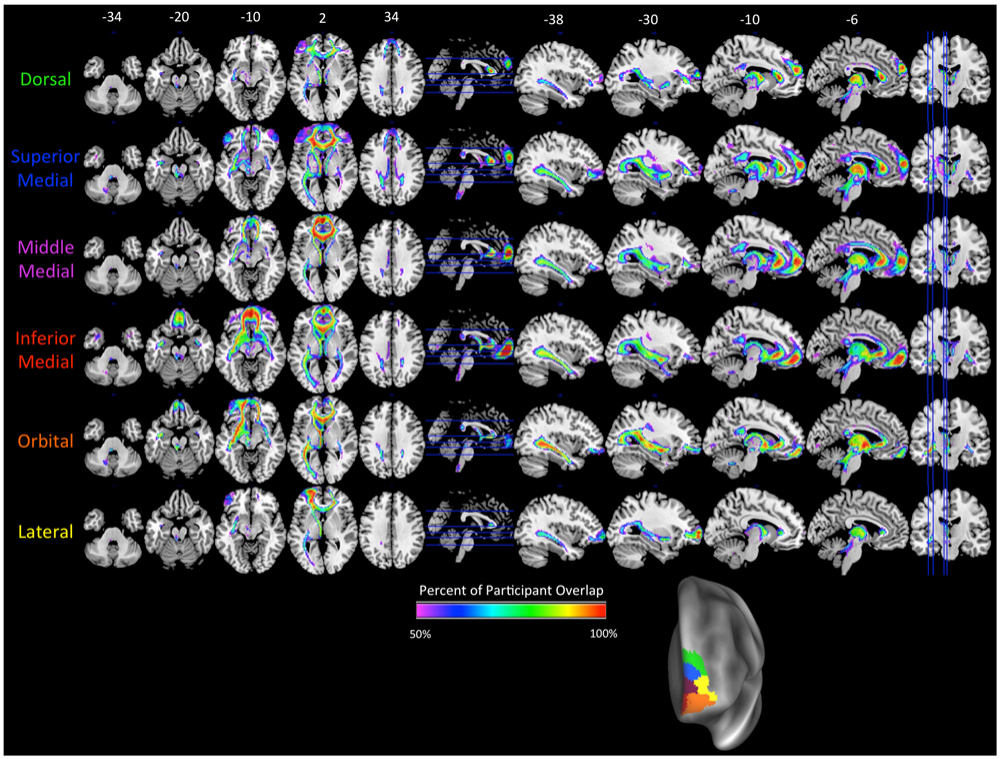
Tracts originating from each of the clusters in the left hemisphere *K* = 6. Label colors (on left) match the cluster colors in Fig 2 (K = 6), which is reproduced at the bottom right. The color scale on brain maps represents the proportion of tract overlap across the participants, with the maps thresholded at 50%. Axial slices are shown in the left column, with slice locations marked on the sagittal slice with a blue line. Sagittal slices are shown in the right column, with slice locations marked on the axial slice with a blue line. MNI coordinates for the *z*-axis (axial slices) and *x*-axis (sagittal slices) are shown above the top row.

**Fig 4:**
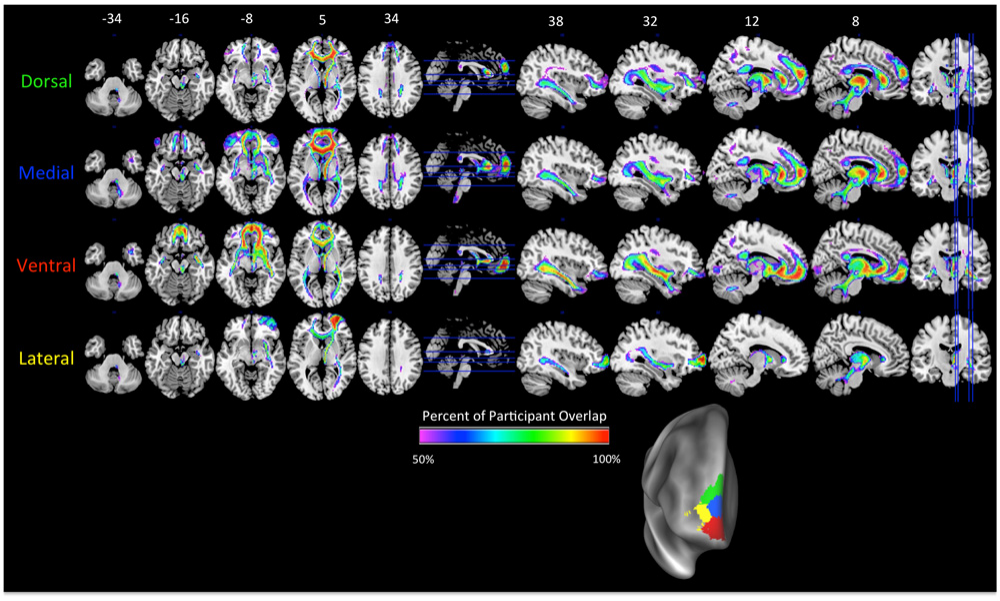
Tracts originating from each of the clusters in the right hemisphere *K* = 4. Label colors (on left) match the cluster colors in Fig 2 (K = 4), which is reproduced at the bottom right. The color scale on brain maps represents the proportion of tract overlap across the participants, with the maps thresholded at 50%. Axial slices are shown in the left column, with slice locations marked on the sagittal slice with a blue line. Sagittal slices are shown in the right column, with slice locations marked on the axial slice with a blue line. MNI coordinates for the *z*-axis (axial slices) and *x*-axis (sagittal slices) are shown above the top row.

**Fig 5:**
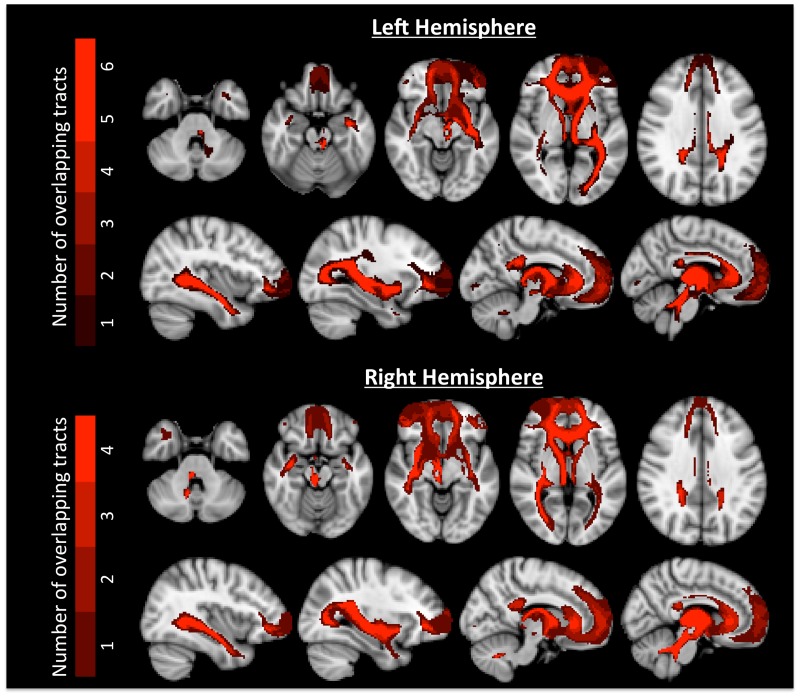
Figure showing the number of clusters that are connected to fiber tracts for the left *K* = 6 solution (top) and the right *K* = 4 solution (bottom). Color bars are shown on the left, with darker red indicating lower overlap and brighter red indicating greater overlap. Most cluster tracts had some degree of overlap with other cluster tracts.

Maps of all of cluster solutions that were evaluated are shown in Fig 2, and coordinate of the center-of-gravity for each cluster are reported in Table 1. At low values of K, a primarily dorsal-ventral gradient was observed, with a medial-lateral gradient also emerging at *K* = 4 for the right hemisphere and *K* = 5 in the left hemisphere. The tracts seeded from each cluster of the left hemisphere, *K* = 6 solution are shown in Fig 3, and tracts seeded from each cluster of the right *K* = 4 solution are shown in Fig 4. Fig 5 shows, for these various white matter tracts, the number of BA10 clusters to which they connect. From these figures, it is evident that the tracts identified in our analysis as connecting to our clusters represent major fiber tracts in the brain. In particular, the inferior fronto-occipital fasciculus (iFOF), uncinate fasciculus (UF), and anterior thalamic radiation (ATR) were connected, at least to some degree, to all of the clusters. The cingulum bundle (CB), however, connected only to medial clusters.

**Fig 6:**
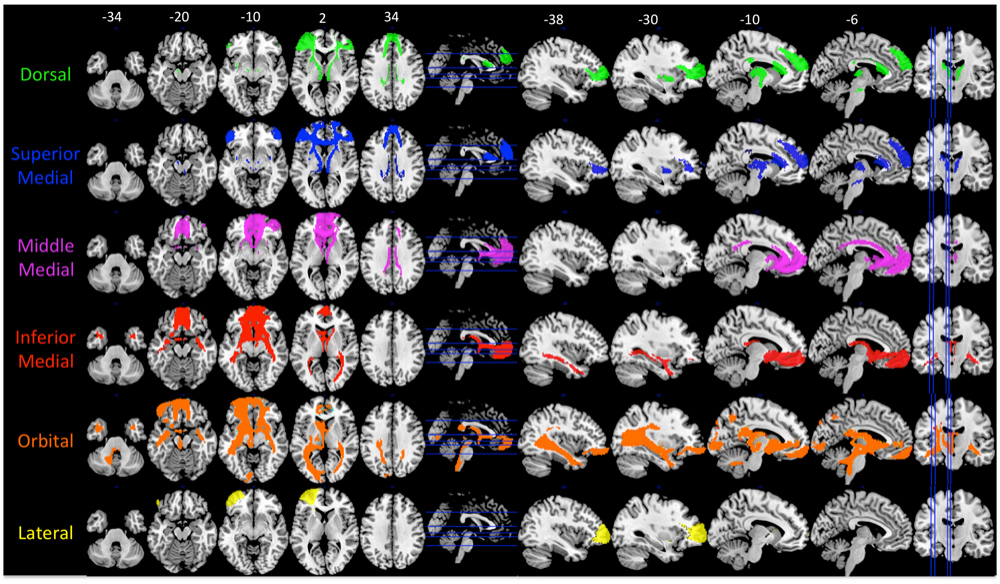
Tracts that had significantly greater connectivity strength to a designated cluster compared to the average of all other cluster tracts for the left hemisphere *K* = 6 solution. Comparisons were cluster-level FWE-corrected to *p*<.01. The color of the tract and the label corresponds to the color of the cluster in Fig 2. Axial slices are shown in the left column, with slice locations marked on the sagittal slice with a blue line. Sagittal slices are shown in the right column, with slice locations marked on the axial slice with a blue line. MNI coordinates for the *z*-axis (axial slices) and *x*-axis (sagittal slices) are shown above the top row.

**Fig 7:**
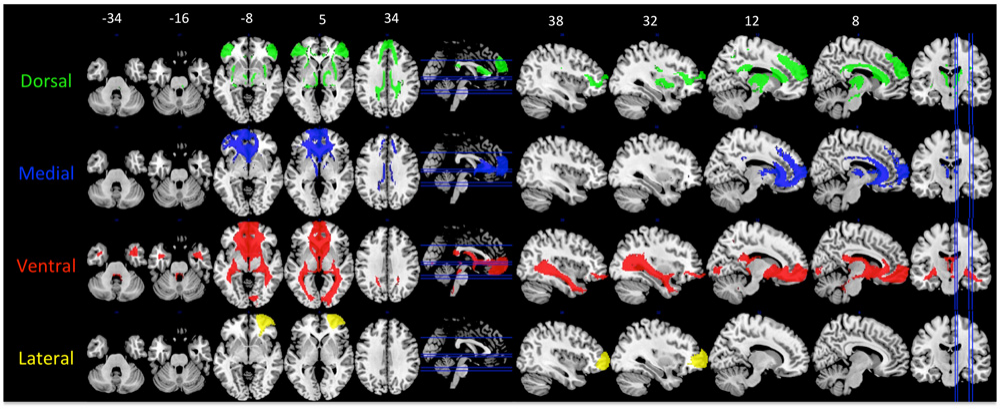
Tracts that had significantly greater connectivity strength to a designated cluster compared to the average of all other cluster tracts for the right hemisphere *K* = 7 solution. Comparisons were cluster-level FWE-corrected to *p*<.01. The color of the tract and the label corresponds to the color of the cluster in Fig 2. Axial slices are shown in the left column, with slice locations marked on the sagittal slice with a blue line. Sagittal slices are shown in the right column, with slice locations marked on the axial slice with a blue line. MNI coordinates for the *z*-axis (axial slices) and *x*-axis (sagittal slices) are shown above the top row.

As the clustering algorithm assigned the clusters based on different patterns of connectivity, one possibility is that the clusters differed in their connectivity strength. To investigate this possibility, we compared the mean connectivity strength of each cluster with the mean connectivity of the other clusters, separately for the left *K* = 6 solution and the right *K* = 4 solution. The results for the left hemisphere are shown in Fig 6, and for the right hemisphere in Fig 7. In the left hemisphere, there was evidence for a dorsal-ventral gradient, as well as limited evidence for a medial-lateral gradient. The more dorsal clusters (i.e., Dorsal, Superior Medial, and Lateral) showed stronger bilateral short-range connections to the Inferior Frontal Gyrus (IFG) than the other clusters, with the level of the IFG termination shifting more ventrally as the cluster moved more ventrally (i.e., from superior medial to dorsal). The ventral-most clusters (i.e., Inferior Medial and Orbital) showed stronger long-range connections to the amygdala and temporal pole via the UF as compared to the other clusters. Connections to the cingulate via the CB were present for the dorsal-medial clusters (i.e., Dorsal, Superior Medial, and Middle Medial), but not the ventral-medial or lateral clusters. Connections to visual cortex via the iFOF were strongest with the Orbital cluster, and to a lesser degree, the Inferior Medial cluster.

The cluster tracts in the right *K* = 4 solution showed a similar pattern as those in the left *K* = 6 solution. The Dorsal and Medial clusters showed stronger connectivity to the IFG than the other clusters, with the Dorsal cluster connecting to more dorsal aspects of the IFG than the Medial cluster. Only the Medial cluster showed connectivity to the cingulate cortex via the CB. The strongest connections to the iFOF and UF were observed for the Ventral cluster. And finally, like in the left *K* = 6 solution, the Lateral cluster had stronger connections to nearby regions of the lateral PFC than other clusters. Thus, although the clusters contributed in an overlapping manner to numerous tracts, the connectivity strength differed between the clusters in a systematic manner. The dorsal-ventral and medial-lateral gradients observed in the clusters themselves were also present in the pattern of connections to white matter tracts.

**Fig 8:**
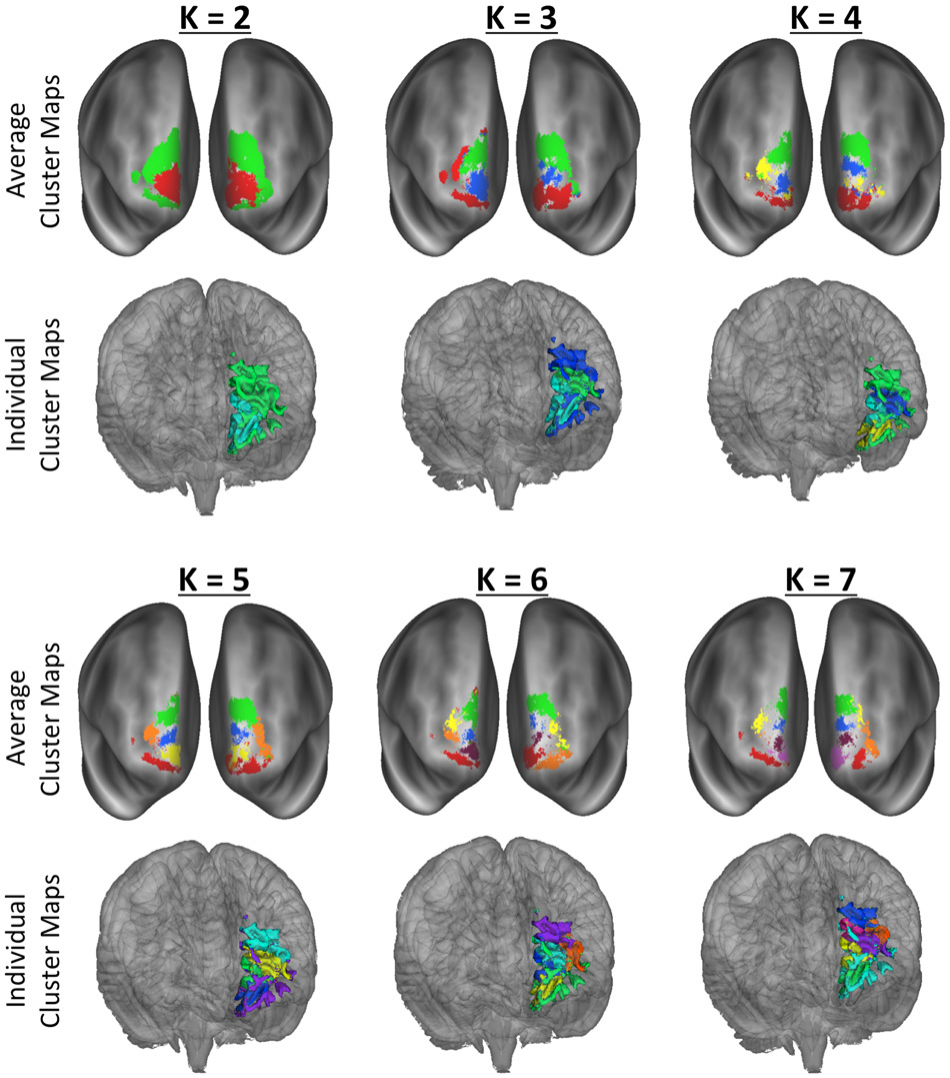
Cluster maps for all clustering solutions for the frontal pole as defined by the Bludau cytoarchitectonic atlas with data from 30 participants from the Human Connectome Project. The Average Cluster Maps were created by dilating the surface maps for each subject with a 2mm spherical kernel, and averaging the resulting maps. The averaged maps shown here were thresholded to only show voxels where the cluster was present in at least 50% of the participants. Colors in the average cluster maps correspond to the closest cluster in Fig 2. The Individual Cluster Maps show the undilated cluster maps from one representative participant (as noted in the text, the undilated surfaces are difficult to average together). The images are from an off-center anterior view in order to show the medial surfaces. Colors in the individual maps do not correspond to the average cluster maps due to graphics software limitations.

**Fig 9:**
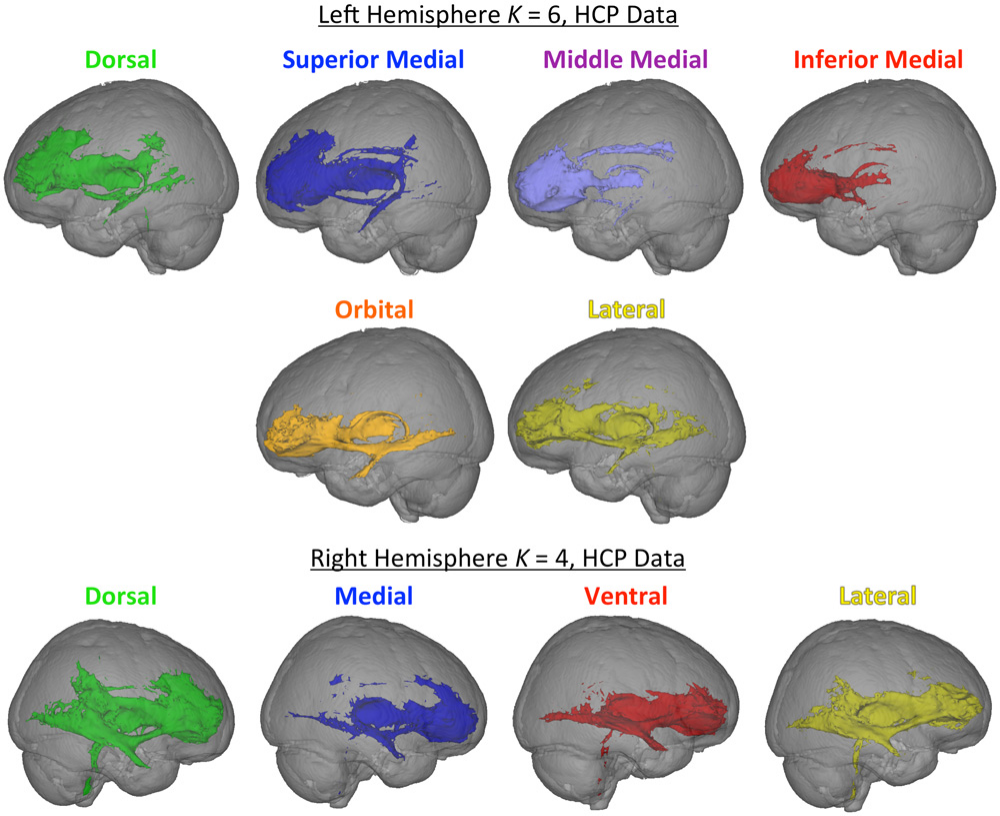
Tracts originating from each of the cluster in the left *K* = 6 solution (top) and the right *K* = 4 solution (bottom) for the Human Connectome Project data. Tract colors match the cluster colors in Fig 8 (average cluster maps). Tracts connecting to these clusters were present in at least 33% of the participants.

In large part, the findings using our data were supported by analysis of the HCP data. As shown in Fig 8, the clusters had a similar organization, with a dorsal-ventral distinction at low values of *K*, and a medial-lateral organization also emerging at higher values of *K*. We then examined which tract connected with each cluster in the left *K* = 6 cluster solution and the right *K* = 4 solution, in order to provide a direct comparison with the above findings. The HCP data also showed a high degree of cluster overlap, as shown in Fig 9 The UF connected to most of the cluster tracts in both the left *K* = 6 and the right *K* = 4 solutions. In line with our data, the medial tracts were connected to cingulate cortex via the CB, and the ventral tracts were connected to visual cortex via the iFOF.

### Functional Co-Activation

**Fig 10:**
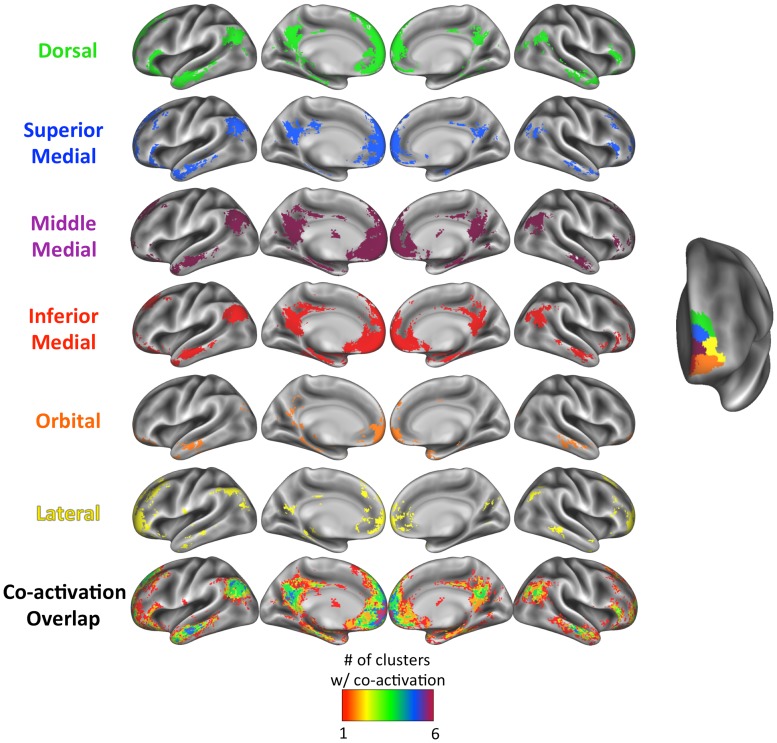
Meta-analytic functional co-activation maps for each cluster of the left *K* = 6 solution. The colors match the cluster labels in Fig 2 (*K* = 6), reproduced on the right. The degree of overlap in the co-activation maps in shown in the bottom row, with the color scale for the overlap shown at the bottom of the figure.

We aimed to confirm previous findings of a medial-lateral distinction in terms of functional co-activation patterns [12,15] and to investigate whether functional co-activation varied along the ventral-dorsal axis as suggested by our data. Functional co-activation maps for each of our clusters as derived from Neurosynth are shown in Figs 10 (for the left FP) and 11 (for the right FP), with the cluster peak coordinates reported in S1 Table (for the left FP) & S2 Table (for the right FP). As shown in the bottom row of these figures, there was a good deal of overlap in the co-activation pattern across the clusters, for both hemispheres. In line with previous meta-analyses and functional connectivity parcellation studies [12,19,34], medial FP clusters co-activated with regions typically thought of as belonging to the default mode network, such as posterior cingulate cortex, temporoparietal junction (TPJ), and subgenual ACC. In addition, the medial clusters were co-activated with inferior frontal gyrus extending to posterior lateral orbitofrontal cortex, as well as the amygdala. Lateral clusters were co-activated with other regions, from the TPJ to the inferior parietal lobule, and from inferior frontal gyrus to dorsolateral prefrontal cortex, in line with suggestions that the lateral FP is more strongly connected functionally to cognitive control regions [12]. While all of the clusters in the right FP showed co-activation with the hippocampus, hippocampal co-activation in the left FP was most evident in ventral medial clusters, and not at all in the lateral and orbital clusters.

**Fig 11:**
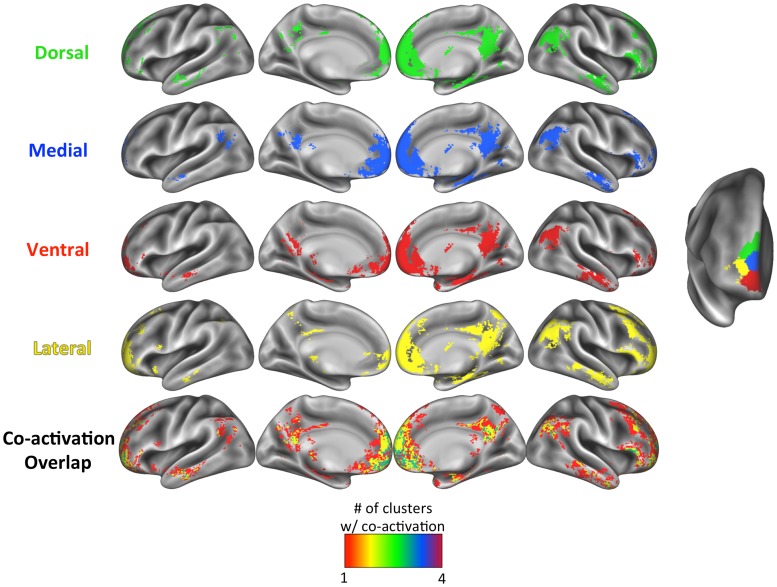
Meta-analytic functional co-activation maps for each cluster of the right *K* = 4 solution. The colors match the cluster labels in Fig 2 (*K* = 4), reproduced on the right. The degree of overlap in the co-activation maps in shown in the bottom row, with the color scale for the overlap shown at the bottom of the figure.

## Discussion

In the current study we used patterns of DTI tractography to parcellate the human FP into distinct subregions. We used a data-driven clustering algorithm to identify the locations of different clusters within the FP, and interrogated the anatomical connections of these clusters with the rest of the brain as derived from DTI data in a large sample of close to 100 individuals, as well as a smaller, confirmatory sample of ultra-high resolution imaging data from the Human Connectome Project. Previous animal and human work has suggested that the FP can be divided in to two or three subdivisions: a medial and lateral portion, with the third area an inferior/ orbital portion. Our findings generally supported these previous findings with some noteworthy exceptions that we detail below. To our knowledge this is the first large-scale DTI parcellation of this human brain region. The size of our sample (n = 93) allowed us to examine the variability in brain organization that hasn’t been possible in previous small-scale examinations of DTI connectivity (n ≈ 10).

### Parcellation Results

We selected the optimal number of clusters (i.e., *K*) based on the VI metric, which reflects the stability of the clustering solution between different groupings [31]. While other metrics may be used to determine the optimal *K* (e.g., silhouette metric), the VI metric is a common metric for evaluating clustering solutions with brain imaging data [32,33,37]. For the left hemisphere, a *K* of 6 was chosen, as the VI metric decreased relative to the VI metric for *K* = 5. Using the same logic, a *K* of 4 was chosen for the right hemisphere. Both solutions resulted in a ventral-to-dorsal and medial-to-lateral arrangement of clusters.

Two other studies have now shown DTI-based parcellations of the FP, but both of these used much smaller samples. Furthermore, the definition of BA 10 varies from ours. Liu and colleagues [19] used the Harvard-Oxford atlas’s demarcation of the FP and found evidence for 3 subregions of FP, while Moayedi and colleagues [34] used BA 10 from the PALS atlas and found evidence for 2 subregions. Both these studies found high symmetry between the left and right hemispheres. In contrast, we used a recently defined cytoarchitectonic atlas from Bludau and colleagues to define BA 10. This atlas divides the FP in to a medial (Fp2) and lateral portion (Fp1) [15]. The Harvard-Oxford FP corresponds fairly well to the FP mask derived from Bludau and colleagues combined Fp1 and Fp2 mask, but extends more laterally and more dorsally. BA 10 from the PALS atlas extends somewhat more laterally than the FP mask from Bludau and colleagues and does not extend ventrally in to the anterior aspects of BA 11. Other cytoarchitectonic atlases besides Bludau and colleagues also extend BA 10 ventrally to BA 11 [16].

Despite using a different definition of FP, our study had some agreement with previous studies in that we found a lateral and medial distinction. Moreover, despite labeling their 3 clusters Lateral, Medial, and Orbital, the clusters of Liu and colleagues [19] clearly have a ventral-to-dorsal gradient, and their lateral cluster corresponds much more closely to the dorsal clusters in our parcellation than the lateral clusters in our parcellation. Moayedi and colleagues [34] did not parcellate the ventral FP, so it is more difficult to compare their results to the current results or to Liu and colleagues [19]. Furthermore, prior studies have only examined limited parcellations. Liu and colleagues [19] only examined *K*-values ranging from 2 to 5, and Moayedi and colleagues [34] only examined *K*-values of 2 and 3. As such, our results provide a more fine-grained picture of parcellation of BA 10, which was possible due to our larger sample size. Furthermore, using ultra-high resolution multi-shell diffusion imaging from the HCP database, we confirmed our results that the FP clusters had a medial-lateral as well as a dorsal-ventral gradient of organization. Nonetheless, despite the differences in the cluster segmentation, there are notable similarities across these three studies in terms of the tractography from the FP, which is discussed below.

### Cluster Connectivity

While connectivity-based parcellations can be useful for identifying subregions within a larger brain region, the parcellation results alone do not reveal information about the underlying connections driving the parcellation. One of the main goals of this study was to gain insight in to the functional organization of the frontal pole by examining its connections. Further, we were interested in directly assessing whether the FP shows a similar medial-lateral and ventral-dorsal organization similar to the PFC as a whole [17]. To this end, we examined the connectivity of each cluster.

The pattern of connectivity observed here replicates aspects of the pattern of connectivity observed in monkeys [18,38] as well as in previous human study of frontal connectivity with many fewer participants [19,34]. In agreement with Liu and colleagues [19], we found that the ventral FP was connected to the amygdala and hippocampus via the UF. However, they found no connections between any cluster and the visual cortex via the iFOF. Indeed, they found no connections with posterior cortex, in line with previous animal work [18]. While the authors themselves did not discuss connectivity to the visual cortex, Fig 5 of Moayedi and colleagues suggests that all of their clusters (i.e., the medial and lateral cluster in a *K* = 2 parcellation and the medial, lateral, and rostral cluster in a *K* = 3 parcellation) had connections with visual cortex via the iFOF, which is line with our initial finding of a high degree of overlap in the tracts that each cluster was connected to. Indeed, one notable aspect of our study was to directly compare the connectivity strength of all of the clusters in order to account for this overlap. This approach was fruitful in that it indicated that the more ventral clusters showed stronger connections to the iFOF compared to the dorsal and lateral clusters.

Moayedi and colleagues found that the lateral FP connected to superior frontal gyrus, extending posteriorly to the superior parietal cortex, however, their figures do not show any tracts leading to these regions. In contrast, the lateral and dorsal FP differentially sent projections locally to superior frontal gyrus, but these tracts did not extend to parietal cortex. Indeed, any connections to the parietal cortex from the FP would pass through the superior longitudinal fasciculus (SLF), which was not identified by Moayedi and colleagues. When we lowered the threshold for the cluster tracts from 50% of participant overlap, we observed the SLF in only about 20% of participants. This suggests that the FP connects to the SLF via neighboring PFC regions such as DLPFC/ frontal eye fields or IFG that have connections to the SLF [39]. Suggesting that such two-step connections exist, the functional coactivation maps from Neurosynth indicated that these regions all functionally co-activated with lateral FP.

One of the more novel aspects of our results was that we observed direct connectivity between the ventral FP and posterior sensory cortex, a pattern not observed in monkeys [18,38]. In monkeys, the most posterior connection that has been observed is with the superior temporal sulcus. One possible explanation for this cross-species difference is that the iFOF has not been observed in monkeys [40,41], with some authors debating its existence in humans. It has been suggested that previous reports of an iFOF in humans may be due to DTI artifacts from neighboring UF and other tracts [40]. However, this argument is at odds with recent anatomical dissection work in humans [42–44]. Performing a careful dissection of the frontal terminations of the iFOF, Sarubbo and colleagues [43] identified 3 components of the deep layers of the iFOF, one of which (the anterior component) terminated in the FP and portions of the basal orbitofrontal cortex which have been classified as being part of the frontal pole [15,16]. These authors confirmed these findings *in vivo* using DTI of one participant. More recently, in a recent tractography study of 20 human participants, Caverzasi and colleagues [44] identified 4 independent subcomponents of the iFOF, with one subcomponent connecting inferior FP and lateral OFC to extra-striate and fusiform visual cortex. Hence, it is not surprising that in our tractography analyses the iFOF was most concentrated in ventral clusters of FP.

### Implications for Functional Organization of the Frontal Pole

**Fig 12:**
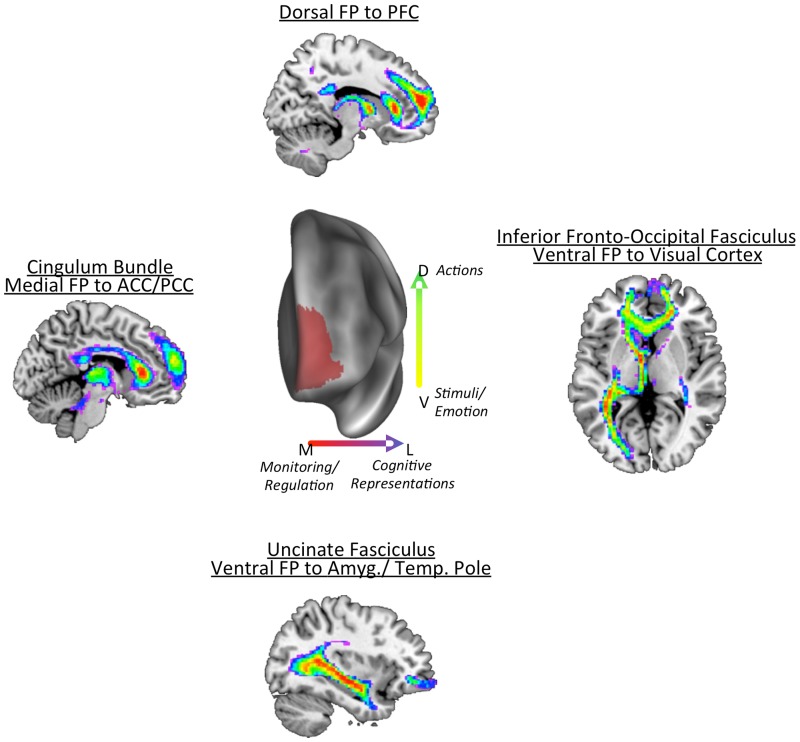
Proposed organizational framework for the frontal pole. We propose that the frontal pole has both a medial-lateral gradient of function as well as a ventral-dorsal gradient of function. Medial areas of FP integrate information from regions involved in monitoring behavior and regulating emotion. Lateral areas (particularly dorsal lateral) connect to posterior PFC regions and to parietal cortex. As such, dorsal lateral areas are involved in maintaining cognitive representations and action plans. Ventral FP connected to regions thought to be involved in linking stimuli to values and emotions.

One of the goals of this study was to glean some insights in to the functional organization of the FP by examining its connectivity, primarily structural connectivity, but also functional co-activation. In Fig 12 we present a framework of the function organization discussed below. We found evidence for both a medial-lateral gradient of function as well as a ventral-dorsal gradient of function. Such an organization exists in the PFC as a whole [17] so it is not surprising that this PFC region, frontal pole, would also show such as organization. As discussed above, the gateway hypothesis posits that the medial and lateral FP differ in terms of their focus of control, with the medial portion exerting control related to external information and the lateral portion exerting control related to internal information [9]. Our results are somewhat at odds with this conclusion, particularly regarding the medial FP controlling external information. We found that anatomically the ventral FP (both medial and orbital) is directly connected to visual cortex. Previously, it had been concluded that the human FP had no connections with posterior sensory cortex, as there was no evidence of such connections in monkeys [18] or in human functional co-activation studies [12]. Supporting a connection from PF to visual cortex, we found that ventral medial and orbital FP co-activate with lateral occipital cortex (BA39/ Angular Gyrus), and furthermore, the ventral orbital FP co-activates with extrastriate and fusiform cortex. Extrastriate and fusiform cortex are the purported posterior targets of the iFOF [43]. *Therefore*, *we propose that the ventral frontal pole is well positioned anatomically to guide attention and behavior related to linking stimuli to values and emotions*.

We also observed commonly identified medial-lateral differences in functional co-activation, with the the medial FP connecting with the cingulate cortex via the CB. The CB appeared to connect medial FP to posterior cingulate, a conclusion confirmed by functional co-activation between the two areas. The posterior cingulate appears to be important for attentional control, particularly in conditions of cue-based anticipatory bias and/or motivation [45,46]. The medial FP also co-activates with pregenual ACC, a region implicated in the regulation of emotion [47]. *Therefore*, *we propose that the medial frontal pole is well positioned anatomically to guide external control based on stimulus-based motivational information*.

The lateral dorsal region of FP connects with lateral prefrontal brain regions, such as DLPFC, that have been implicated in maintaining and updating goals. In this manner, the most dorsal region appears to be involved in internally directed control, consistent with the gateway hypothesis [48]. Researchers have shown that the dorsal and lateral FP are activated in prospective memory tasks, where the participant must carry out a pre-determined action after a delay often involving an intervening task [48]. Here, the action plan must be maintained internally in order to respond appropriately. This region has also been implicated in ‘branching’ control, or determining the appropriate action by integrating past and present task information [49]. Further, this region has been shown to be activated by conditions where participants voluntarily choose between different tasks in a random order [50–52]. Such situations require participants to keep track of their recent task choices to compare to an abstract mental model of randomness. *As such*, *we propose that the dorsal lateral section of the FP is well positioned anatomically to guide attention and behavior related to the overall goals and plans*, *connecting with regions that store and update current task information*.

## Conclusions

The connectivity patterns of the different FP clusters suggest a framework for a functional organization of the frontal pole based on three levels of processing: affective, monitoring, and goal-directed processing. As current functional models of FP organization only describe differences between the medial and lateral portion, the current anatomical work provides a framework for future research examining potential dissociations between these regions in patterns of activation. Understanding how the FP incorporates affective and cognitive information may provide crucial insights in to illnesses characterized by poor reward-based decision making such as drug addiction [53]. More generally, contrasting with previous human work with far fewer participants (i.e., less than 35) [19,34], the current study involving 93 participants, demonstrates the benefit of connectivity studies with larger numbers of participants.

## Supporting Information

S1 Table Peaks from clusters showing significant functional co-activation with seeds in the left frontal pole. Coordinates of cluster peaks are in MNI space. When clusters covered multiple regions, local maxima are reported. Brodmann areas were obtained via the Talairach daemon. BA: Brodmann Area, vox: number of voxels in cluster.
(XLSX)

S2 Table Peaks from clusters showing significant functional co-activation with seeds in the right frontal pole. Coordinates of cluster peaks are in MNI space. When clusters covered multiple regions, local maxima are reported. Brodmann areas were obtained via the Talairach daemon. BA: Brodmann Area, vox: number of voxels in cluster.
(XLSX)
